# Extraction of saponins from soapnut and their application in controlling ammonia and particulate matter

**DOI:** 10.1039/d5ra03653d

**Published:** 2025-08-19

**Authors:** Changwon Chae, Jiseok Hong, Hyunjung Kim, Dong Hyun Kim, Seung Oh Lee, Ijung Kim

**Affiliations:** a Department of Civil and Environmental Engineering, Hongik University Seoul 04066 Republic of Korea ijung.kim@hongik.ac.kr +82-43-649-1779 +82-43-649-1335

## Abstract

Synthetic surfactants have been associated with environmental concerns, requiring their replacement with natural alternatives such as saponins. In this study, saponin extraction from soapnut was optimized using the reflux method through the Box–Behnken design and response surface methodology (RSM). Foams generated from the saponin solution were evaluated for effectiveness in ammonia and particulate matter (PM) removal, along with foam characteristic analysis. The optimized saponin extraction yielded 30.18% saponin, exceeding the yields achieved through conventional methods. The extracted saponin exhibited enhanced foamability with increasing concentrations, leading to an expanded surface area that facilitated the removal ammonia and PM removal through foam-based adsorption. Ammonia removal was further improved as the foamability and stability of surfactant foams increased at saponin concentrations above the critical micelle concentration (CMC) under neutral pH conditions. The comparable PM removal efficiency of saponin-based foams to that of synthetic surfactants underscores their potential as an effective and environmentally sustainable solution for air pollution control.

## Abstract 2

Foams generated from soapnut-derived saponins can be utilized for the removal of air pollutants such as ammonia and particulate matter.

## Graphical abstract

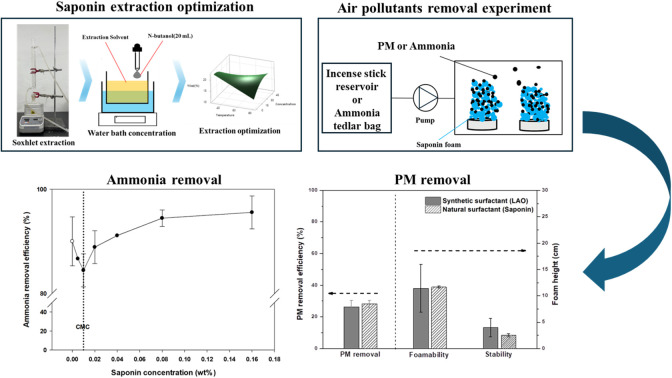

## 1 Introduction

Surfactants, essential in a variety of industrial applications, have primarily been derived from petrochemical sources. These petroleum-based surfactants often exhibit significant toxicity and limited biodegradability, posing risks to the environment, which has the potential to exert toxic effects on aquatic and terrestrial species, including humans.^1,2^ In response to these challenges, industries have increasingly adopted eco-friendly, highly biodegradable natural surfactants in applications such as liquid detergent production,^3^ emulsion formulation,^4^ and enhanced oil recovery.^5^ Comparative analyses demonstrated that natural surfactants like saponin extracted from soapnut, offered competitive foamability and stability compared to synthetic alternatives.^6,7^ However, despite their ecological advantages, natural surfactants faced challenges such as lower yields and the necessity for optimized extraction processes.^8,9^

Given the environmental benefits of natural surfactants, researchers have worked to improve their extraction techniques, with two primary optimization methods currently in use. The first approach, the one-factor-at-a-time (OFAT) method, systematically adjusted a single variable at a time to refine surfactant extraction processes.^10,11^ The second, the response surface method (RSM), employed regression analysis to develop equations that explained the relationships between variables and yields, facilitating the identification of optimal conditions.^12,13^ However, many studies that focused on enhancing the extraction efficiency of natural surfactants neglected to assess other critical properties, such as foam characteristics and their potential applications in contaminant removal.

Air pollutants such as ammonia, have been recognized as precursors to particulate matter (PM), contributing to air quality deterioration and posing significant health risks.^14,15^ Especially, the majority of ammonia emissions in the United States, approximately 60%, were attributed to livestock manure, while 20% originated from synthetic fertilizers.^16^ Once released into the atmosphere, ammonia reacted with pollutants such as SO_*x*_ and NO_*x*_, forming secondary PM and further exacerbating air pollution. Given its role as a PM precursor, ammonia contributed to indirect health risks by increasing ambient PM concentrations, which had been associated with cardiovascular and pulmonary diseases. The combined health risks presented by PM and ammonia underscore the necessity for effective removal strategies to mitigate both direct exposure hazards and further air pollution.

Ammonia and PM have conventionally been removed using packed towers, spraying, biological treatment, or zeolite adsorption.^17,18^ These methods were characterized by complex equipment requirements, reliance on chemicals, and high operational costs, thereby necessitating more efficient and cost-effective removal approaches.^19,20^ Considering the increased adsorption surface area is essential for enhancing removal efficiency, the utilization of surfactant solutions to generate foams represents an effective approach, as the foams continuously offer an expanded interface for pollutant capture.^21–23^ Previously, synthetic surfactant foams were applied to capture coal dust during mining, achieving a removal efficiency of over 70%.^24,25^ Also, dust suppression using synthetic surfactant foams achieved a reduction in dust generation exceeding 30% and a decrease in water usage of more than 70% relative to conventional spraying methods.^26^ However, research on the potential of natural surfactants for air pollutant removal remains limited.

In this study, the extraction process of saponin from soapnut was optimized using a Box–Behnken design and RSM to enhance saponin yield. Furthermore, the saponin foam-based removal of ammonia and PM, along with its foam characteristics, was examined to determine its potential as an environmentally friendly alternative to synthetic surfactants in air pollution control.

## 2 Materials and methods

### 2.1. Extraction and quantification of saponin

Commercially available dry soapnut husks were grounded to a particle size below 75 μm and subsequently mixed with 200 mL of ethanol and/or deionized water mixture in a three-neck flask for extraction. Saponin was extracted using the reflux extraction method,^12^ under varying extraction temperature (30 to 80 °C), ethanol concentration (0 to 50%), soapnut-to-solvent ratio (0.04 to 0.1 g mL^−1^), and extraction time (1 to 9 hours).

The saponin extract was subjected to vacuum filtration and subsequently concentrated in the water bath at 100 °C to enable saponin quantification. The dried extract was dissolved in 10 mL of deionized water, followed by the addition of 20 mL of 99% *n*-butanol to precipitate the saponins. The resulting mixture was dried again at 100 °C to evaporate the *n*-butanol, thereby concentrating the extract and isolating the saponins.^27,28^ The isolated saponins were dissolved in deionized water and analyzed using a UV-Vis spectrophotometer (METTLER TOLEDO, UV5) at a wavelength of 425 nm. Calibration curves were determined using a saponin standard (CAS no. 8047-15-2) in the concentration range of 0.5 to 4 mg mL^−1^. The extraction yield was then calculated using the following equation.1
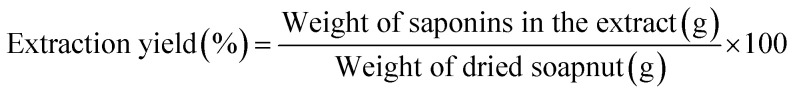


### 2.2 Optimization of extraction process

The effects of four variables (extraction temperature, ethanol concentration, soapnut-to-solvent ratio, and extraction time) on extraction yield were evaluated through single-factor experiments. Subsequently, the Box–Behnken design and RSM were applied to optimize the extraction process.^29–31^ A total of 27 experimental runs were performed, incorporating four independent variables selected based on their significant influence on saponin yield. The statistical significance was evaluated through analysis of variance (ANOVA). Saponin yield was defined as the dependent variable, and a second-order polynomial equation was developed to describe its relationship with the independent variables.

2 Yield(%) = *a*_0_ + *a*_1_*A* + *a*_2_*B* + *a*_3_*C* + *a*_4_*D* + *a*_5_*A*^2^ + *a*_6_*B*^2^ + *a*_7_*C*^2^ + *a*_8_*D*^2^ + *a*_9_*AB* + *a*_10_*AC* + *a*_11_*AD* + *a*_12_*BC* + *a*_13_*BD* + *a*_14_*CD*

where *a*_i_ represents the regression coefficients, and each term corresponds to the main effects, interactions, and quadratic terms. *A*, *B*, *C*, and *D* denote extraction temperature (*A*), ethanol concentration (*B*), soapnut-to-solvent ratio (*C*), and extraction time (*D*), respectively.

### 2.3 Characterization of saponin solution

The extracted saponin was dissolved in deionized water for foam generation, and its foamability and stability were evaluated using the Ross-Miles method (Ross and Miles, 1941). Initially, 50 mL of the solution was placed in the foam receiver. The solution in the 200 mL foam pipette, positioned above the receiver, was then gradually released to induce foam formation. The foamability was determined by measuring the foam height after 200 mL of solution was completely released from the foam pipette. Foam stability was evaluated using *R*_5_ (ref. 7 and 32) and drainage,^4,33^ which was determined by measuring the reduction in foam height or the corresponding increase in solution height, respectively, 5 minutes after foam generation, as follows:3
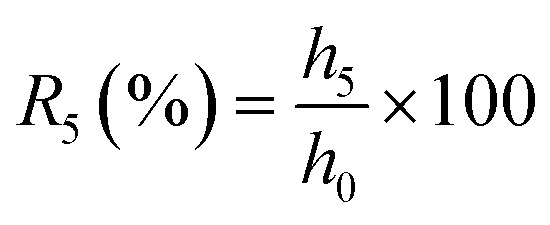

4 Drainage(cm) = *h*_s,5_ − *h*_s,0_

where, *h*_0_ and *h*_5_ represent the foam heights immediately after foam generation and after 5 minutes, respectively, while *h*_s,0_ and *h*_s,5_ indicate the corresponding heights of the saponin solution. Additionally, the viscosity of the saponin solution was measured using an Ostwald viscometer at room temperature, and relative viscosity was calculated based on the viscosity of deionized water (1 cP). The critical micelle concentration (CMC) of the saponin solution was determined based on foamability where the linear slope of foam generation *versus* concentration exhibited a distinct change.

### 2.4 Ammonia removal experiment

Ammonia removal experiments were conducted by introducing the ammonia gas from a Tedlar bag into a rectangular test chamber (length: 47.5 cm × width: 37.5 cm × height: 20 cm) at a flow rate of 0.5 L min^−1^ using a gas pump (QC-10N, SIBATA). Ammonia concentration was monitored using an ammonia gas detector (Ventis Pro, Gastron). To ensure uniform dispersion of ammonia gas within the chamber, a fan was installed at the top. A circular tank (diameter: 18 cm) containing 1 L of saponin solution or deionized water and foam generators (S5, SRF) was placed inside the chamber (Fig. S1). Once the ammonia concentration in the chamber reached 100 ppm, the gas injection to the chamber was stopped and the foam generators were activated to generate foams. A series of experiments were conducted under varying solution pH levels (3–11), foam generator flow rates (0.5–2.0 L min^−1^), solution volumes (100–1500 mL), and saponin concentrations (0–0.16 wt%) to identify the optimal conditions for ammonia removal.

Foamability and stability experiments, conducted under the same conditions as the ammonia removal experiments, used a foam generator with a flow rate ranging from 0.5 to 2.0 L min^−1^. A 300 mL of saponin solution was placed in an acrylic cylinder with a diameter of 18 cm and a height of 50 cm. Foamability was assessed by measuring the foam height after 140 seconds of bubble generation, while stability was evaluated by recording the foam height remaining 5 minutes after foam generation was ceased.

### 2.5 PM removal experiment

The PM removal experiment was conducted by placing 3 L of saponin solution in a test chamber (*L*: 50 cm × *W*: 50 cm × *H*: 50 cm), and PM concentrations were measured using an aerosol spectrometer (DustDecoder 11-D, Grimm), following the methodology in a previous study (Fig. S1).^23^ A 1 cm segment of an incense stick was burned to generate PM in a separate reservoir and injected into the test chamber using a gas pump (N96, KNF). Once the PM concentration reached 250 μg m^−3^, the PM injection to the chamber was stopped and foams were generated using a foam generator (KK-180C, Paladin) to initiate the PM removal process. Given that the PM generated from incense combustion was predominantly below 1 μm, this study specifically focused on the removal of PM1.0.

Foamability and stability tests were also conducted as described in Section 2.4. These tests were performed under the same conditions as the PM removal experiments, with the flow rate of the foam generator set at 5 L min^−1^.

### 2.6 Removal efficiency calculation

#### 2.6.1 Ammonia removal

Ammonia removal efficiency was assessed by comparing two experimental configurations designed to isolate the effect of saponin-generated foam. The first configuration, referred to as the blank test, involved introducing ammonia gas into an empty acrylic chamber that contained only the ammonia sensors and four foam generators, without any saponin solution. Air bubbling was maintained for 60 minutes to simulate the experimental conditions. This setup allowed for the quantification of ammonia losses due solely to passive adsorption onto the chamber walls and internal apparatus, without interference from liquid-phase or foam-mediated interactions. The second configuration, the foam test, was conducted under identical conditions, except that 1 L of 0.16 wt% saponin solution was added to the chamber. As foam was generated continuously for 60 minutes, the remaining ammonia concentration reflected the combined effects of gas–liquid absorption and foam interaction. By comparing the ammonia concentrations measured at the end of the 60-minutes period for both conditions, the specific removal efficiency attributable to the saponin foam could be determined, while correcting for background adsorption effects. The ammonia removal efficiency was calculated using the following equation:5
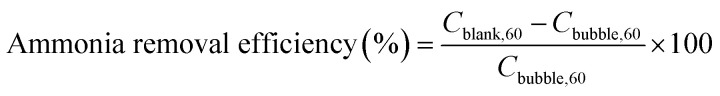
where *C*_blank, 60_ is the ammonia concentration remaining after 60 minutes of air bubbling without foam, and *C*_bubble, 60_ is the ammonia concentration remaining after 60 minutes of foam generation using the saponin solution.

#### 2.6.2 PM removal

The removal efficiency of particulate matter (PM) was evaluated using a mass balance approach within a continuous flow system, in which incense sticks were used as the PM emission source.

Three experimental configurations were implemented to accurately determine the amount of PM captured by the saponin-generated foam. First, in the air blank test, PM was introduced into an empty chamber without any saponin solution, while the foam generator operated for 60 minutes. This setup quantified background losses attributable to natural deposition onto the chamber walls and the foam generator. Second, in the bubble blank condition, foam was produced using the saponin solution without PM injection. This test measured mist particles generated from foam collapse, which could otherwise be misidentified as PM by the aerosol spectrometer (DustDecoder 11-D, Grimm). Third, in the bubble test, PM was introduced into the chamber containing the saponin solution, and foam was continuously generated for 60 minutes to assess the removal performance of the foam.

To calculate the net PM removal efficiency, the total PM introduced into the system was adjusted by subtracting several components: background losses from the air blank test, mist particle interference measured in the bubble blank test, and the cumulative PM mass detected as discharged through the aerosol spectrometer during the experiment. In addition, the residual PM concentration within the chamber at the end of the bubble test was measured and deducted. This comprehensive correction ensured that the final PM removal efficiency reflected only the amount of PM effectively captured by the saponin-generated foam. The PM removal efficiency was calculated using the following equation.^23^6
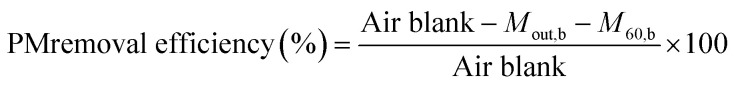
where air blank refers to the net PM introduced into the chamber after accounting for background losses (*i.e.*, air blank = *M*_in_ − *M*_c_, where *M*_in_ is the total PM mass injected into the chamber, and *M*_c_ is the background PM mass measured during blank tests, representing natural deposition onto chamber walls and equipment surfaces without any treatment). *M*_out,b_ is the cumulative PM mass detected as discharged by the aerosol spectrometer, and *M*_60,b_ is the residual PM mass suspended in the chamber at the end of the test.

## 3 Results and discussion

### 3.1. Single factor experiments

**Fig. 1:**
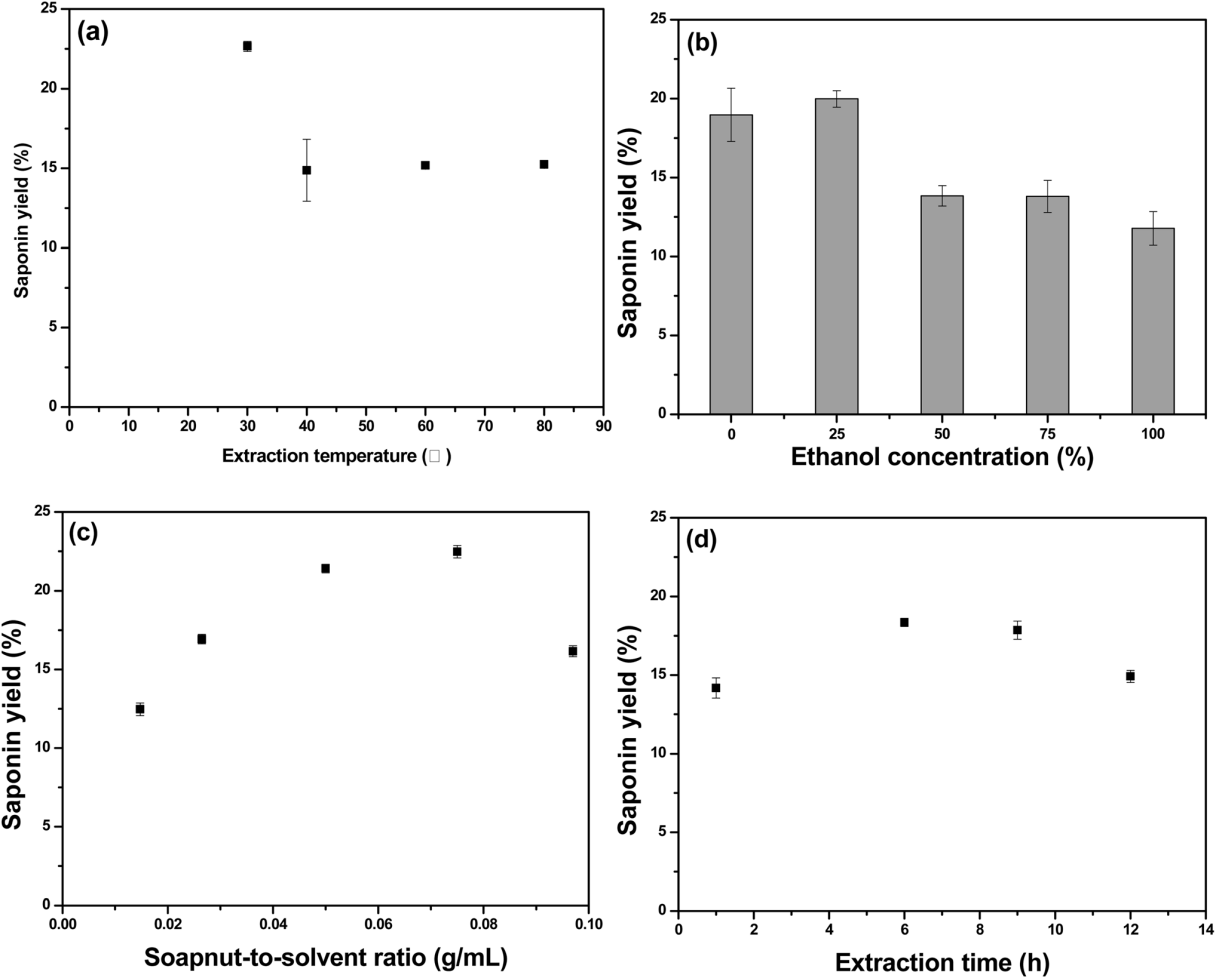
Effect of (A) extraction temperature, (B) ethanol concentration, (C) soapnut-to-solvent ratio, and (D) extraction time on extraction yield.

Single-factor experiments were conducted to examine the effects of extraction temperature, ethanol concentration, soapnut-to-solvent ratio, and extraction time on saponin yield and to establish the experimental range for each variable in the Box–Behnken design (Fig. 1).

Regarding the effect of temperature, the highest saponin yield was recorded at 30 °C, after which the yield declined with increasing temperature. This trend was likely attributed to the heat-sensitive nature of the molecular structure of saponin, which was prone to degradation at elevated temperatures (50–70 °C) as reported in previous studies.^34,35^

The effect of ethanol concentration on saponin extraction indicated that yields were highest at concentrations below 25%, with a notable decline observed beyond this threshold (Fig. 1B). This trend was strongly associated with solvent polarity, as bioactive compounds like saponin dissolve effectively in polar solvents such as water or diluted ethanol, whereas their solubility decreases in less polar solvents like pure ethanol.^36,37^ Additionally, elevated ethanol concentrations induced protein coagulation in plant extracts, further hindering saponin recovery.^38^ Since ethanol concentrations exceeding 50% reduced the yield, solvents with ethanol concentrations below 25% were considered to have polarity more compatible with that of saponin.

The relationship between the soapnut-to-solvent ratio and saponin yield demonstrated an optimal point at 0.075 g mL^−1^, beyond which the yield was declined. This finding was consistent with previous studies,^10,39,40^ which indicated that excessive powder concentration could reduce solubility and facilitate the extraction of unwanted compounds, such as polysaccharides and proteins, ultimately lowering saponin recovery.

Regarding extraction time, saponin yield steadily increased up to 6 hours, after which it remained stable until 10 hours before slightly decreasing at 12 hours. This result suggests that the extraction process reached its maximum efficiency at around 6 hours, with no further improvement beyond this point. The minor decline at 12 hours was likely due to saponin degradation or reduced purity caused by the dissolution of impurities such as polysaccharides and proteins.^41^

### 3.2 Optimization of extraction variables

The predictive model developed through ANOVA estimated a maximum yield of 30.18% under optimal conditions of an extraction temperature of 30 °C, ethanol concentration of 0%, a soapnut-to-solvent ratio of 0.04 g mL^−1^, and an extraction time of 1 hour. The desirability score was calculated to be 1.0000, suggesting that the model achieved near-optimal performance with no further refinements required.^42,43^

A regression model was established based on the actual measurements obtained from the experimental series detailed in Table S1, with the resulting regression equation as below.

7 Yield(%) = 56.18 − 0.10*A* − 0.50*B* − 642*C* − 0.70*D* − 0.0027*B*^2^ + 3233*C*^2^ + 0.0032*AB* + 3.05*BC* + 0.0179*BD*

The residuals were calculated using the parameter values and the regression equation for each of the 27 experiments (Table S2). The regression model yielded an *R*^2^ value of 0.8338, and ANOVA confirmed its statistical significance with an *F*-value of 9.48 and a *P*-value <0.001 (Table 1), indicating the reliability of the model. Furthermore, the actual measurements from the designed experiments closely corresponded to the predicted values generated by the regression model, yielding an *R*^2^ value of 0.9879 (Fig. S2). As shown in Table 2, these findings indicate an improved extraction yield compared to previous optimization studies employing conventional methods such as maceration,^44,45^ soxhlet,^45,46^ and reflux.^40,47^

**Table 1:** ANOVA for the regression model

Source	Degree of freedom	*F*-value	*P*-value
Model	9	9.48	<0.001
Temperature	1	0.52	0.480
Ethanol concentration	1	33.08	<0.001
Soapnut-to-solvent ratio	1	24.66	<0.001
Extraction time	1	2.19	0.157
Ethanol concentration × ethanol concentration	1	3.34	0.085
Soapnut-to-solvent ratio × soapnut-to-solvent ratio	1	9.69	0.006
Temperature × ethanol concentration	1	2.88	0.108
Ethanol concentration × soapnut-to-solvent ratio	1	3.74	0.070
Ethanol concentration × extraction time	1	2.29	0.148

**Table 2:** Saponin yields from different extraction techniques and plant sources

Extraction method	Materials	Extraction time (h)	Saponin yield (%)	References
Maceration	Azadirachta excelsa	1	13.30	Letchumanan *et al.*, 2024
	Chromolena odorate	120	18.10	Fauzi *et al.*, 2020
Soxhlet	Gleditsia peel	24	31	Nhat Do *et al.*, 2019
	Chromolena odorate	12	21.60	Fauzi *et al.*, 2020
Reflux	Panax notoginseng	1.52	31.96	Hu *et al.*, 2018
	Camellia oleifera	1	9.44	Yu *et al.*, 2023
	Sapindus mukorossi (soapnut)	1	30.18	This study

To assess the purity of the extracted saponins, UV absorbance analysis was conducted using a test solution prepared by dissolving the dried crude extract in water at a concentration of 7 mg mL^−1^. The UV-based quantification revealed a saponin content of 1.6903 mg mL^−1^, indicating that approximately 24.15% of the crude extract consisted of saponins. This result provides a reliable estimate of the saponin purity under the optimized extraction conditions (Table S3). The specific types of saponins present in soapnut extracts can be identified through analytical techniques such as HPLC-MS and FT-IR. High-Performance Liquid Chromatography (HPLC) enables the separation of individual saponin compounds based on their retention time and polarity, while Fourier Transform Infrared Spectroscopy (FT-IR) provides information on characteristic functional groups such as hydroxyl, carbonyl, and glycosidic linkages, which are common in triterpenoid saponins.^48,49^ Previous studies have identified four oleanane-type triterpenoid saponins in *Sapindus mukorossi* extracts, namely Mukurozisaponin E1, Rarasaponin II, Mukurozisaponin G, and Rarasaponin VI.^50,51^

### 3.3 Saponin foam characteristics

**Fig. 2:**
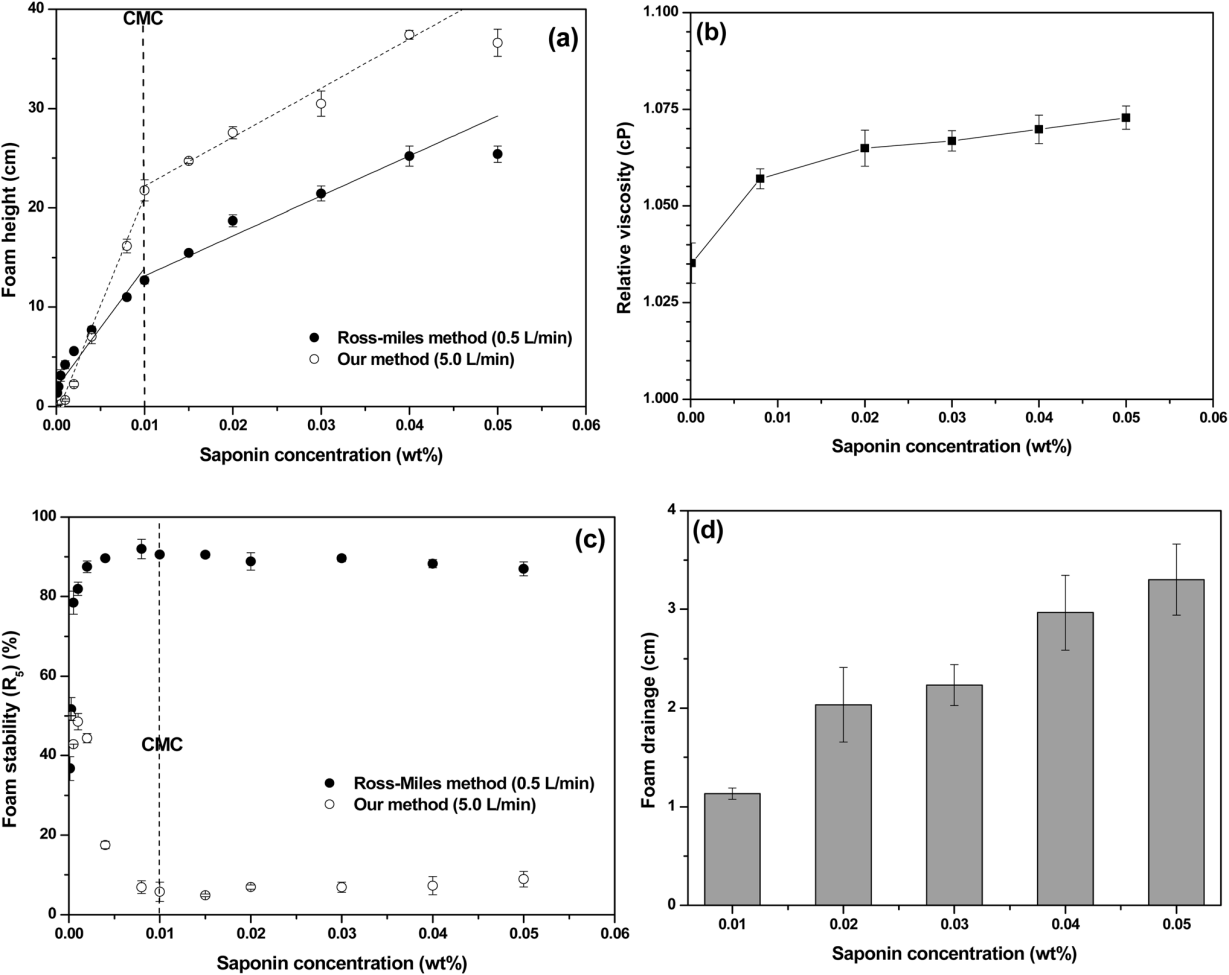
Foam characteristics at different saponin concentrations: (A) foamability, (B) viscosity, (C) stability, and (D) drainage.

Evaluating the foam properties using the Ross-Miles method across various saponin concentrations revealed that foamability increased steadily with increasing saponin concentrations and saturated beyond 0.04 wt% (Fig. 2A). Notably, the slope of the linear increase in foamability changed significantly at 0.01 wt%, which was correlated with a decrease in stability and a reduced increase in viscosity. Especially, the viscosity of the solution was increased rapidly up to 0.01 wt% (Fig. 2B). Foam stability based on *R*_5_ values, exceeded 50% at saponin concentrations above 0.000125 wt%, peaked at 0.01 wt%, and then slightly declined with further increases in concentration (Fig. 2C). This behavior was attributed to reduced foam drainage due to the sharp increase in viscosity up to 0.01 wt%, which enhanced stability.^52^ However, at concentrations exceeding 0.01 wt%, the increased presence of surfactant molecules in the lamellae might lead to heavier foam, thereby accelerating foam drainage and reducing stability (Fig. 2D). The change in the linear trends of foamability and viscosity, along with the foam stability, indicates that this region corresponds to the CMC of the solution,^53–55^ suggesting that the CMC of the extracted saponin was 0.01 wt%.

As shown in Fig. S3, foamability tests conducted under the same conditions as the ammonia removal experiments revealed that foamability increased with the flow rate, a trend attributed to the larger volume of air introduced into the saponin solution, which promoted greater foam generation. The effect of pH variations on foamability showed that foam generation was fairly vigorous under neutral conditions.^56,57^ Furthermore, foamability was steadily increased with saponin concentration up to 0.04 wt%, beyond which it was plateaued. A strong correlation was observed between the foamability and stability of the saponin solution. As foam stability improved, the foam retention time was prolonged, enabling greater foam accumulation and consequently enhancing foamability. However, at higher flow rates, the correlation between foamability and stability weakened. Although increased flow rates facilitated greater air injection and foam formation, the associated rise in shear forces likely generated less stable foams with thinner films, resulting in reduced foam stability.

Both Fig. 2 and S3 showed that foamability increased with rising saponin concentration under both 2.0 and 5.0 L min^−1^ flow rates. Foam stability at 2.0 L min^−1^ followed a trend consistent with the Ross-Miles method (0.5 L min^−1^), however, at 5.0 L min^−1^, stability decreased with increasing concentration, converging at 0.01 wt%. This suggests that elevated foam generation flow rates under overflow conditions may diminish foam stability.^58^ Additionally, foam stability at 0.08 wt% was declined at flow rates above 0.5 L min^−1^ (Fig. S3B), likely due to the foamtion of less stable foams under higher shear conditions.^59^ These differences in foam characteristics, depending on the foaming method, highlight the importance of utilizing not only the Ross-Miles method commonly used to evaluate surfactant solution properties, but also SI assessments that reflect the specific foaming conditions.^60^

### 3.4 Ammonia removal using saponin-based foams

**Fig. 3:**
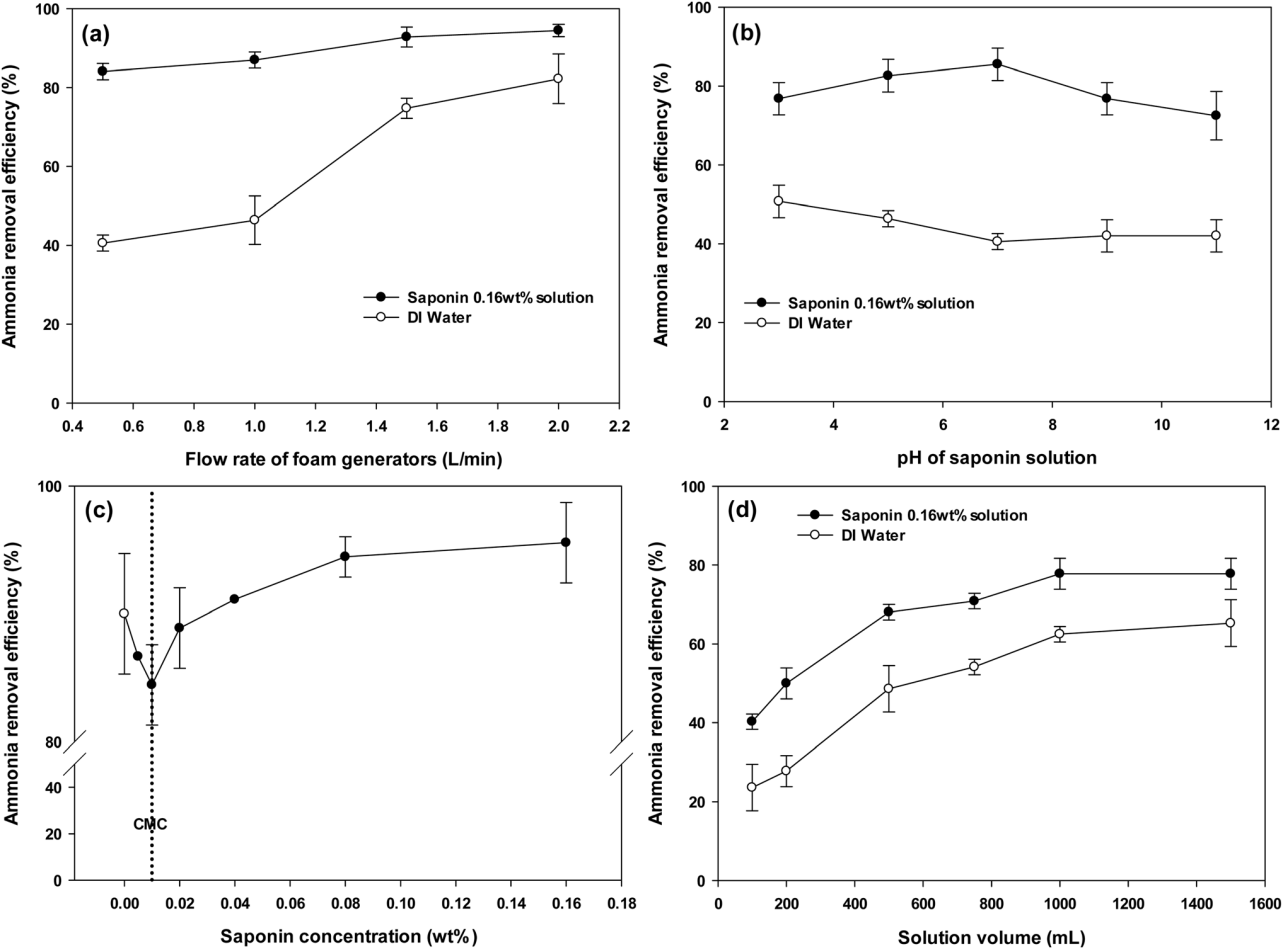
Change in ammonia removal efficiency by (A) flow rate of foam generators, (B) pH of saponin solution, (C) saponin concentration, and (D) saponin solution volume.

Ammonia removal experiments using saponin solution revealed that the removal efficiency increased with higher foam generation flow rates, saponin concentrations, and solution volumes at pH 7 (7.08 ± 0.08) (Fig. 3). The highest removal efficiency was observed at pH 7, with a decline noted as pH deviated from neutrality, reaching a minimum at pH 11 (Fig. 3B). This trend can be explained by the acid–base equilibrium of ammonia (p*K*_a_ ≈ 9.25),^61^ where at neutral pH, ammonia predominantly exists as the water-soluble ionized form (NH_4_^+^), which can be effectively retained in the foam's aqueous phase. However, under alkaline conditions (pH > 9.25), the equilibrium shifts toward the uncharged form (NH_3_), which, although partially remaining dissolved, exhibits higher volatility and lower solubility compared to NH_4_^+^.^62^ This makes it less likely to be retained in the foam matrix, thereby reducing the overall removal efficiency. In addition, the removal efficiency of saponin was lower under acidic conditions, likely because foam generation was more effective under neutral conditions.^63^ Under identical conditions using deionized water, higher ammonia removal efficiency was observed under acidic conditions. This can be attributed to the fact that ammonia exists predominantly in the form of ammonium ions in acidic environments.^64^ Furthermore, ammonia removal efficiency did not exhibit a linear relationship with increasing saponin concentration. Instead, the efficiency initially decreased up to 0.01 wt%, followed by a gradual increase, reaching a maximum at 0.16 wt%. This trend suggests that as the saponin concentration approached its CMC of 0.01 wt%, the associated reduction in surface tension may have led to the release of absorbed ammonia into the chamber.^65^ Since surface tension typically reaches a minimum at the CMC, it is reasonable that the surface tension remains unchanged with further increases in saponin concentration beyond this point. As shown in Fig. S3E, the foamability increased continuously up to 0.04 wt%, contributing to improved ammonia removal. These findings imply that maintaining saponin concentrations above the CMC is essential for effective gaseous ammonia removal, ensuring sufficient foam generation. Although foamability plateaued beyond 0.04 wt%, higher saponin concentrations likely promoted micelle formation,^66,67^ thereby effectively trapping dissolved ammonia within the solution and further enhancing removal efficiency. This improvement can be attributed to the combined effects of neutral pH, enhanced foam generation, increased saponin concentration, increased flow rate, and greater solution volume, all of which contributed to expanding the available surface area for ammonia absorption within the chamber.^23^ Additionally, the retention time of ammonia gas was prolonged as the generated foam was sustained, allowing for effective absorption into the saponin solution coating the foam surface.^68^ The difference in removal efficiency between water and saponin solution across varying solution volumes further supported this finding, with saponin solution exhibiting an average removal efficiency improvement of 17.13 ± 3.36%.

### 3.5 PM removal using saponin-based foams

**Fig. 4:**
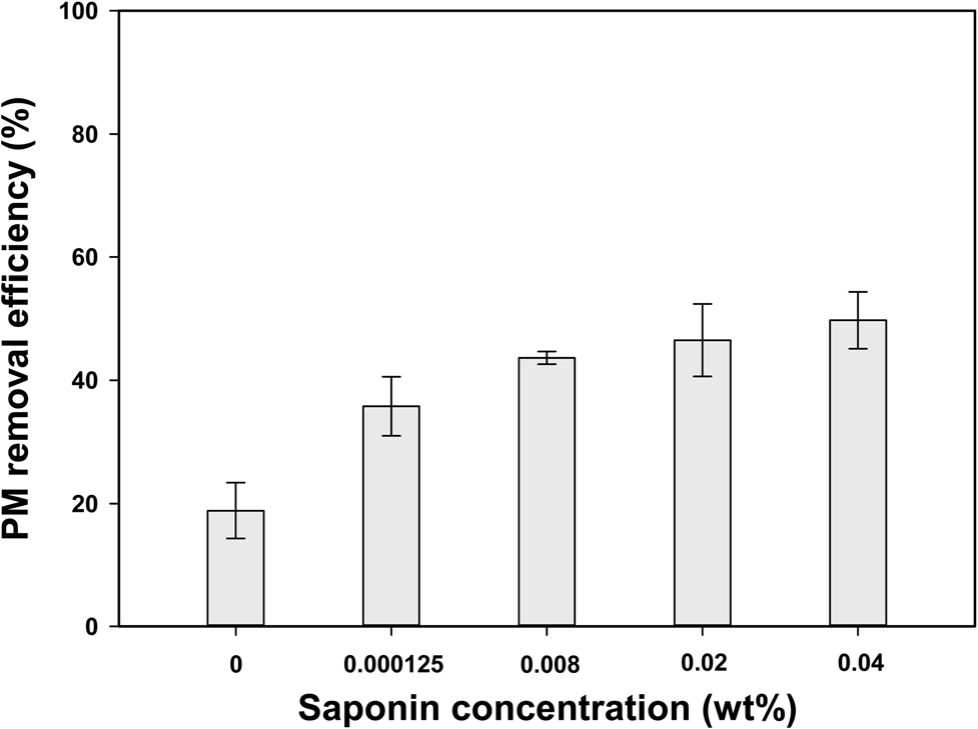
Effect of saponin concentration on PM removal efficiency.

The removal efficiency of PM increased with higher saponin concentrations (Fig. 4), likely due to the reduction in surface tension that enhanced foam formation and, in turn, expanded the available adsorption surface area. A rapid increase in PM removal efficiency was observed when a small amount of saponin (0.000125 wt%) was dissolved in deionized water due to the increased viscosity of the solution (Fig. 2B), thereby enhancing the adsorption of PM from the headspace. Moreover, the rate of increase in removal efficiency decreased, suggesting that the changes in foam formation near the CMC were reflected in the corresponding increase in removal efficiency (Fig. 2A and B). This phenomenon was attributed to the increase in the number of surfactant molecules at the water surface with rising saponin concentrations, eventually reaching saturation at the CMC of 0.01 wt%. These findings highlight a strong interrelationship between foamability, viscosity, and PM removal efficiency.

Although surfactant molecules reached saturation at concentrations exceeding the CMC, the removal efficiency continued to increase. This enhancement was attributed not only to the high concentration of saponin in the solution, but also to the ability of the surfactants to form micelles at concentrations above the CMC as supported by previous studies showing that micelle formation above the CMC facilitated contaminant removal through adsorption mechanisms.^67,69^

**Fig. 5:**
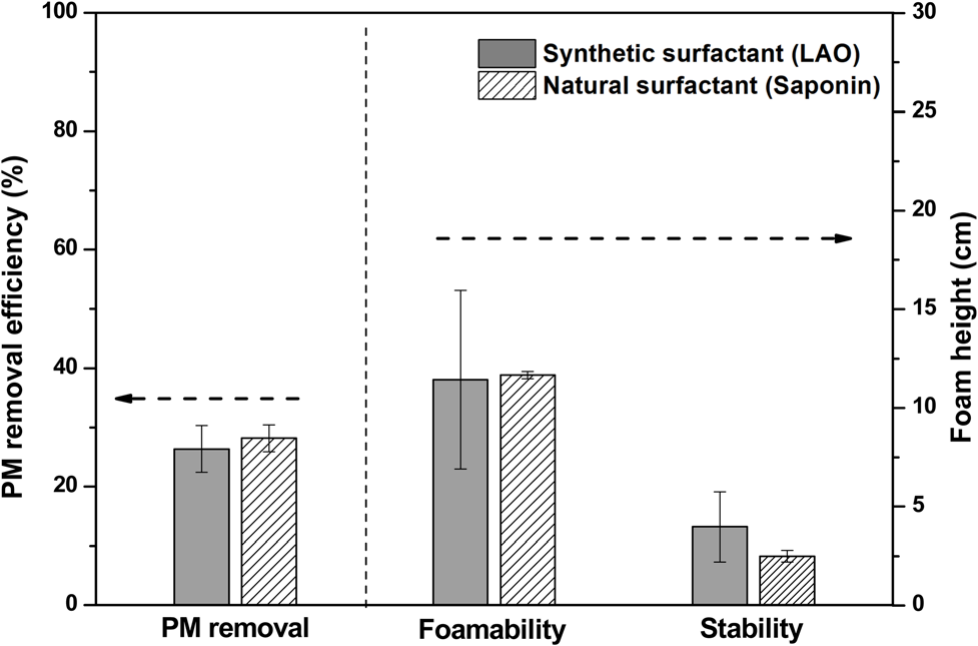
Comparison of synthetic and natural surfactants in PM removal and foam characteristics.

As shown in Fig. 5, the experiments conducted using 0.05 wt% of extracted saponin yielded a removal efficiency of 28.16 ± 2.27%, comparable to that achieved with synthetic surfactants, lauryl amine oxide (LAO).^23^ The foamability of 0.05 wt% saponin was 11.67 ± 0.19 cm, closely aligning with the 11.43 ± 4.52 cm observed for LAO. These results reinforced the correlation between foamability and PM removal efficiency and demonstrated the potential of natural surfactants like saponin as environmentally friendly alternatives for air pollutant removal.

## 4 Conclusions

The extraction of saponin from soapnut was successfully optimized in this study, achieving a high yield of 30.18% under optimal conditions, including an extraction temperature of 30 °C, 0% ethanol concentration, a soapnut-to-solvent ratio of 0.04 g mL^−1^, and a 1-hour extraction time. Using the saponin-based foams, the maximum ammonia removal efficiency was recorded at 98.5 ± 2.12%, alongside a particulate matter (PM) removal efficiency of 49.77 ± 4.63%.

Increased saponin concentrations enhanced foamability, stability, and viscosity. The CMC of the extracted saponin was approximately 0.01 wt%, at which significant changes in foamability, stability, and viscosity were observed. Furthermore, increased air flow rates and saponin concentrations enhanced foam generation, particularly under neutral pH conditions. The trends observed in foam characteristics strongly correlated with ammonia and PM removal efficiency, underscoring the critical role of foam properties in effective air pollutant removal.

The PM removal efficiency of saponin-based foams was comparable to that of synthetic surfactants, demonstrating the potential of saponin as a sustainable and environmentally friendly alternative to synthetic surfactants for air pollution control.

## Author contributions

Changwon Chae: writing – original draft, methodology, investigation, data curation, conceptualization. Jiseok Hong: investigation, validation. Hyunjung Kim: investigation, validation. Dong Hyun Kim: investigation, validation. Seung Oh Lee: writing – review & editing, investigation, validation. Ijung Kim: conceptualization, writing – review & editing, methodology.

## Conflicts of interest

There are no conflicts to declare.

## Supplementary Material

RA-015-D5RA03653D-s001

## Data Availability

All data presented in this study are available upon request. The datasets supporting Fig. 1–5, and S3 are accessible *via* Open Science Framework (OSF) at https://doi.org/10.17605/OSF.IO/S7FZM. Supplementary information is available. Table S1: Experimental design matrix for optimization tests, Table S2: Residual analysis of measured and predicted yields, Table S3: Reproducibility of saponin purity under optimized extraction conditions, Fig. S1: Schematic diagram of ammonia or PM removal experiment, Fig. S2: Comparison of measured and predicted saponin yields, Fig. S3: Foamability (left side) and stability (right side) of saponin-based solutions under varying conditions: (a) and (b) flow rate variation at pH 5 and 0.08 wt% saponin, (c) and (d) pH variation at 0.08 wt% saponin and 0.5 L min^−1^ flow rate, and (e) and (f) saponin concentration variation at 2.0 L min^−1^ flow rate and pH 7. See DOI: https://doi.org/10.1039/d5ra03653d.
