# Supplementary material for: Extraction of saponins from soapnut and their application in controlling ammonia and particulate matter
Source: RSC Adv. 2025 Aug 19;15(36):29190–200. doi: 10.1039/d5ra03653d (PMC12377307; doi:10.1039/d5ra03653d)
Supplement: RA-015-D5RA03653D-s001 [file RA-015-D5RA03653D-s001.pdf]

Supplementary Information (SI) for RSC Advances.
This journal is © The Royal Society of Chemistry 2025

Supplementary Information

Extraction of Saponins from Soapnut and Their Application in Controlling Ammonia and Particulate Matter

Changwon Chae, Jiseok Hong, Hyunjung Kim, Dong Hyun Kim, Seung Oh Lee, Ijung Kim*

Department of Civil and Environmental Engineering, Hongik University, Seoul 04066, Republic of Korea

*Corresponding author. Phone: +82-43-649-1335. Fax: +82-43-649-1779. E-mail: ijung.kim@hongik.ac.kr

Table S1 Experimental design matrix for optimization tests

Test No.	Extraction temperature (℃)	Ethanol concentration (%)	Soapnut-to-solvent ratio (g/mL)	Extraction time (h)
1	30	25	0.07	1
2	55	50	0.07	9
3	30	25	0.1	5
4	55	25	0.1	1
5	30	0	0.07	5
6	80	0	0.07	5
7	80	50	0.07	5
8	55	0	0.1	5
9	55	50	0.07	1
10	55	25	0.07	5
11	80	25	0.04	5
12	30	25	0.07	9
13	55	50	0.1	5
14	55	25	0.07	5
15	55	0	0.07	9
16	55	25	0.1	9
17	55	50	0.04	5
18	80	25	0.07	1
19	30	50	0.07	5
20	55	25	0.04	1
21	30	25	0.04	5
22	55	25	0.07	5
23	55	0	0.07	1
24	55	25	0.04	9
25	55	0	0.04	5
26	80	25	0.1	5
27	80	25	0.07	9

Table S2 Residual analysis of measured and predicted yields

Test No.	Measured yield (%)	Predicted yield (%)	Residual*
1	22.26	20.58	2.814
2	16.84	15.58	1.582
3	9.97	8.72	1.547
4	12.58	11.75	0.688
5	23.61	23.18	0.183
6	14.98	16.39	1.989
7	22.23	21.16	1.152
8	16.41	14.37	4.180
9	20.40	17.37	9.213
10	14.80	16.38	2.496
11	16.26	15.35	0.825
12	11.96	14.36	5.758
13	25.28	26.67	1.944
14	11.28	14.25	8.829
15	17.96	15.31	7.008
16	13.11	12.04	1.154
17	21.35	22.66	1.716
18	25.84	21.67	17.366
19	12.07	15.87	14.489
20	14.38	14.89	0.258
21	19.09	20.88	3.208
22	9.65	9.45	0.039
23	12.91	15.28	5.602
24	10.63	11.01	0.147
25	15.65	15.86	0.048
26	15.05	15.86	0.658
27	16.31	15.86	0.201

*$Residual = (Measured\ yield - Predicted\ yield)^2$

Table S3 Reproducibility of saponin purity under optimized extraction conditions

Test No.	Crude extract concentration (mg/mL)	Saponin concentration measured (mg/mL)	Saponin purity (%)
1	7	1.5663	22.375
2		1.7998	25.711
3		1.7569	25.100
4		1.6381	23.401

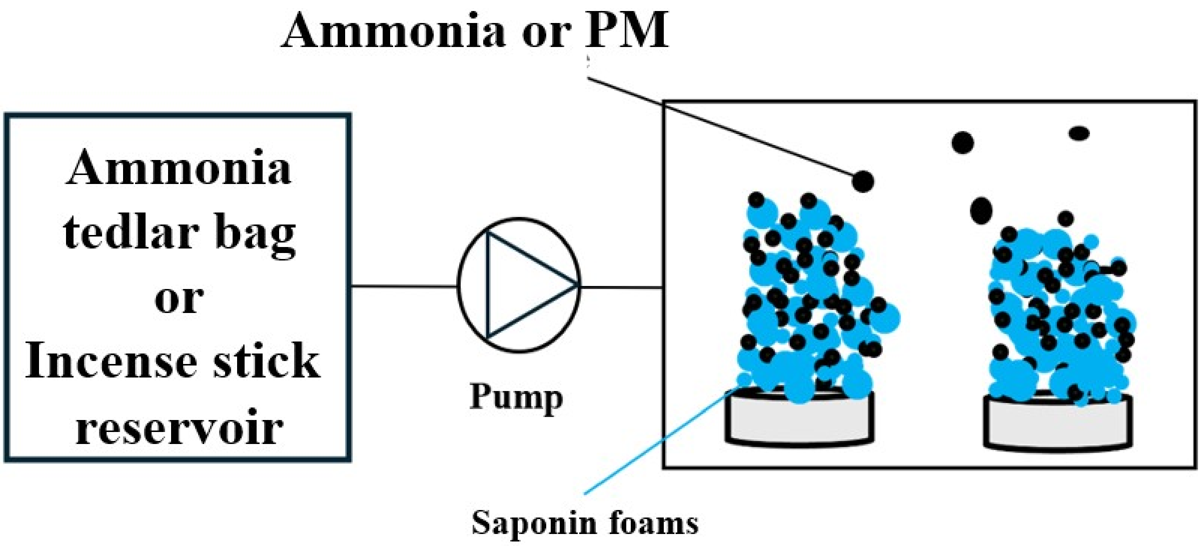

Fig. S1 Schematic diagram of Ammonia or PM removal experiment

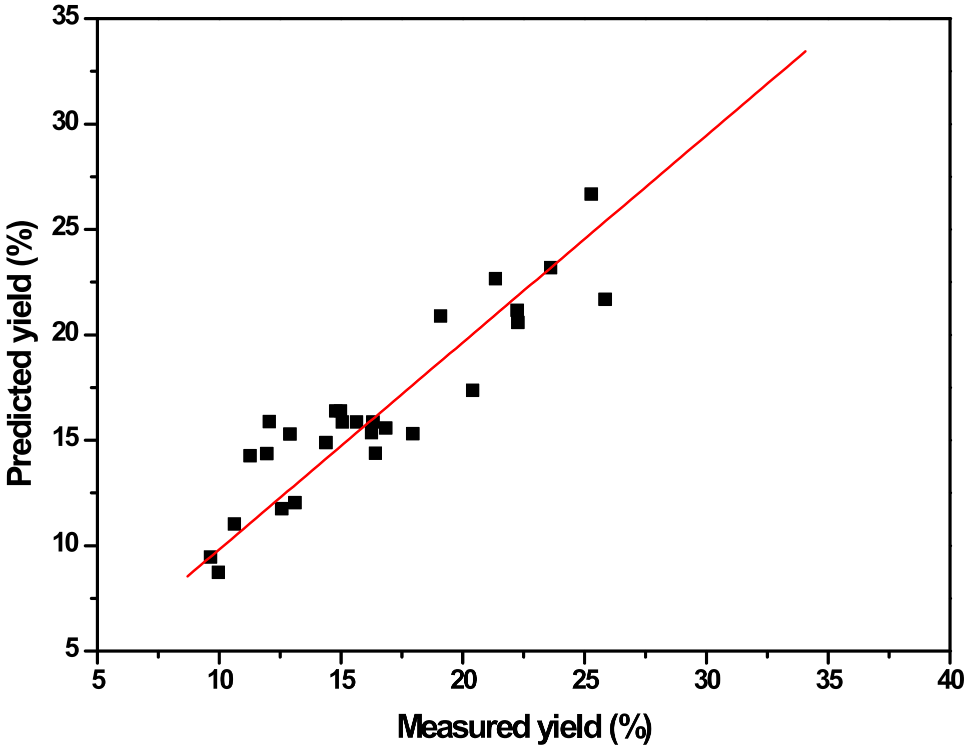

Fig. S2 Comparison of measured and predicted saponin yields

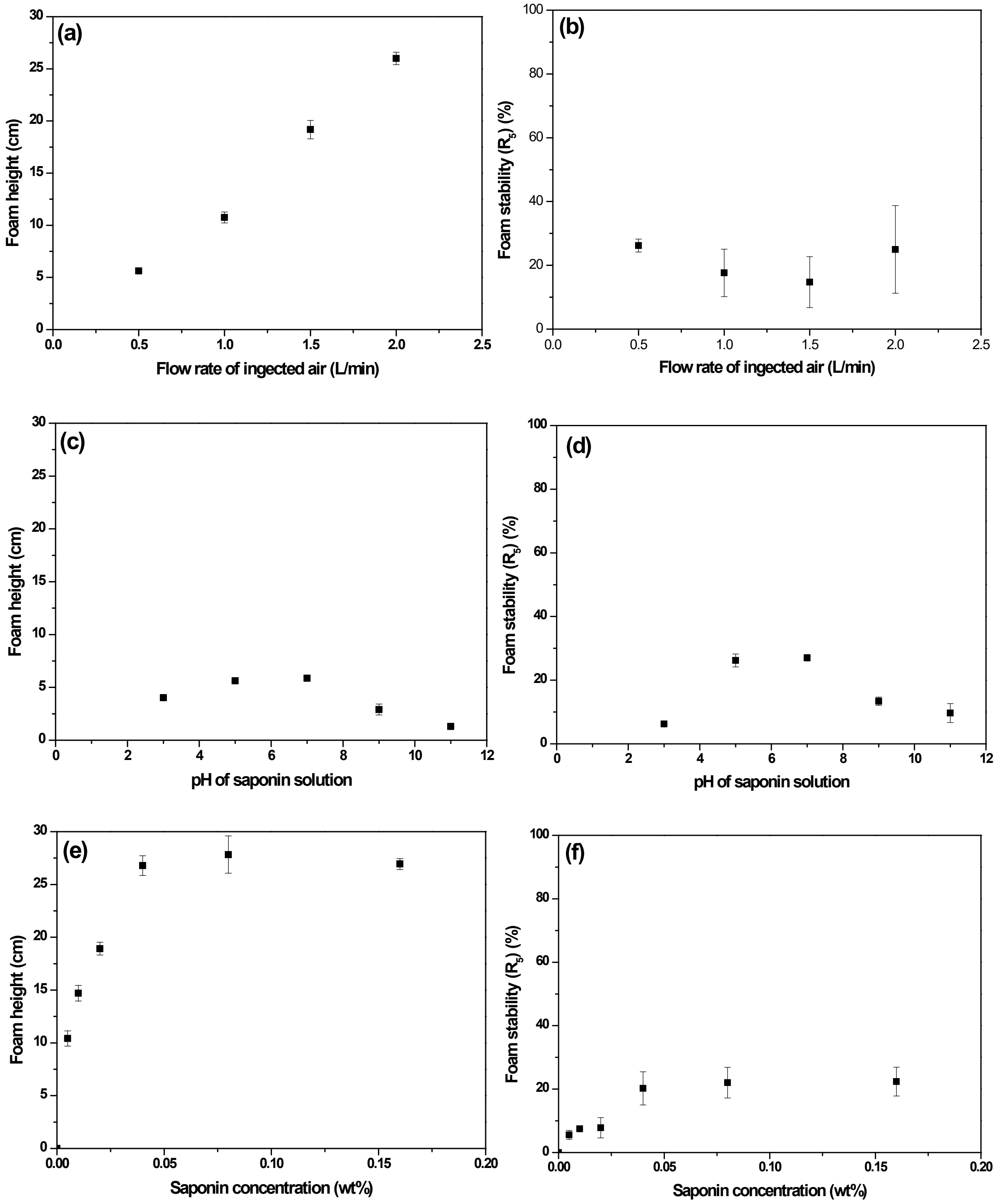

Fig. S3 Foamability (left side) and stability (right side) of saponin-based solutions under varying conditions: (a) and (b) flow rate variation at pH 5 and 0.08 wt% saponin, (c) and (d) pH variation at 0.08 wt% saponin and 0.5 L/min flow rate, and (e) and (f) saponin concentration variation at 2.0 L/min flow rate and pH 7
